# Measurement of distances and locations of thoracic and lumbar vertebral bodies from CT scans in cases of spinal deformation

**DOI:** 10.1186/s12880-024-01293-6

**Published:** 2024-05-14

**Authors:** Alexander T. D. Grünwald, Susmita Roy, Renée Lampe

**Affiliations:** 1 Department of Clinical Medicine, Center for Digital Health and Technology, Klinikum rechts der Isar, Department of Orthopaedics and Sports Orthopaedics, Research Unit of the Buhl-Strohmaier Foundation for Cerebral Palsy and Paediatric Neuroorthopaedics, Technical University of Munich, TUM School of Medicine and Health, Munich, Germany; 2 Markus Würth Professorship, Technical University of Munich, Munich, Germany

**Keywords:** Computed tomography, Spinal deformation, Vertebral body, Anatomical data, Body scanner

## Abstract

**Background:**

Spinal deformations, except for acute injuries, are among the most frequent reasons for visiting an orthopaedic specialist and musculoskeletal treatment in adults and adolescents. Data on the morphology and anatomical structures of the spine are therefore of interest to orthopaedics, physicians, and medical scientists alike, in the broad field from diagnosis to therapy and in research.

**Methods:**

Along the course of developing supplementary methods that do not require the use of ionizing radiation in the assessment of scoliosis, twenty CT scans from females and males with various severity of spinal deformations and body shape have been analysed with respect to the transverse distances between the vertebral body and the spinous process end tip and the skin, respectively, at thoracic and lumbar vertebral levels. Further, the locations of the vertebral bodies have been analysed in relation to the patient’s individual body shape and shown together with those from other patients by normalization to the area encompassed by the transverse body contour.

**Results:**

While the transverse distance from the vertebral body to the skin varies between patients, the distances from the vertebral body to the spinous processes end tips tend to be rather similar across different patients of the same gender. Tables list the arithmetic mean distances for all thoracic and lumbar vertebral levels and for different regions upon grouping into mild, medium, and strong spinal deformation and according to the range of spinal deformation.

**Conclusions:**

The distances, the clustering of the locations of the vertebral bodies as a function of the vertebral level, and the trends therein could in the future be used in context with biomechanical modeling of a patient’s individual spinal deformation in scoliosis assessment using 3D body scanner images during follow-up examinations.

## Background

According to the World Health Organization (WHO), idiopathic and neurogenic scoliosis are among the largest contributors to the need for rehabilitation services in both children and adults [1]. Scoliosis is a condition where the vertebral column is laterally curved by at least 10^∘^ in coronal view in combination with rotation and torsion around the vertical axis [2]. Depending on the cause, scoliosis can occur at all ages and may develop in different degrees of severity. Its prevalence increases from less than 1 % in newborns to 1-2 % in adolescents [3]. In elderly people of the age of 60 to 90 the prevalence ranges up to 68 %, including secondary scoliosis [4, 5]. The high rates in the elderly may be either a continuation of adolescent idiopathic scoliosis, or *de novo* due to degenerative processes and other causes [6]. Depending on the cause and severity of scoliosis and the age of the affected person, different forms of treatment are required, ranging from conservative physiotherapy to surgical intervention [7]. Due to the potentially rapid progression of scoliosis, especially during growth, an early diagnosis and regular follow-ups are important [8]. In addition to the clinical examination, X-rays are the gold standard for initial diagnosis, followed by CT and MRI, if necessary [9]. The lateral curvature of the spine is quantified by measuring the Cobb angle from the X-ray image in coronal view [10]. The state and progression of scoliosis are then usually monitored by regular X-rays. As a result, young patients who show signs of scoliosis are often exposed to significant ionizing radiation from regular X-rays during follow-up examinations, which can be associated with an increasing risk of radiation-related health problems later in life [11, 12]. Therefore, young individuals in particular could benefit from alternative scoliosis assessment methods that are free of ionizing radiation and help to reduce the number of X-rays in follow-up examinations. This could lower the potential risk of long-term radiation related health issues.

**Fig. 1:**
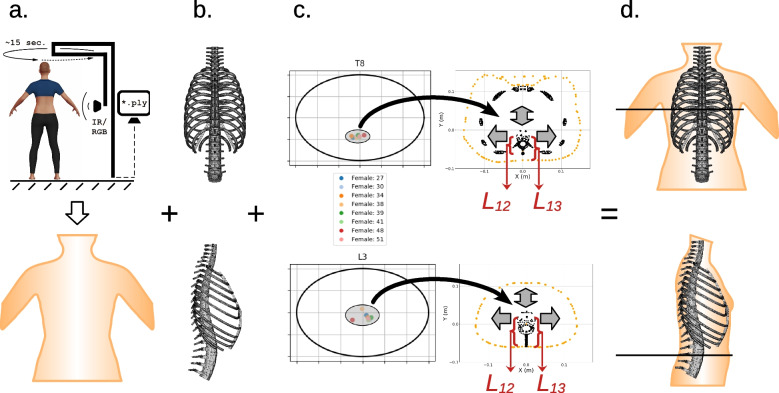
Schematic representation of some potential use of the current results for fitting biomechanical models in the body scan. Patient’s spinal deformation could be assessed by taking a 3D body scan (**a**) and comparing it with simulations on a biomechanical model of the vertebral column and rib cage (**b**). Current results as the location of the cluster could help to align the biomechanical model inside the 3D body scan at certain vertebral levels (**c**). At its best match between the biomechanical model and the 3D body scan, the patient’s individual spinal deformation could then be derived from the corresponding specific biomechanical model simulation (**d**)

Towards an alternative for scoliosis assessment during follow-ups that is free of the use of ionizing radiation, among others [13], methods based on the analysis of 3D body scanner images of the torso have been developed [14–16]. This body scanner uses infrared scanning and video technologies to reconstruct a 3D image of the scanned person’s outer body contour and thus is completely non-invasive and free of ionizing radiation. Moreover, the entire scanning and image reconstruction process is fairly fast and comfortable for the patient [14]. For the analysis of the reconstructed 3D image of the patient’s outer body contour and to further derive the spinal curvature from it, finite-element method (FEM) simulations have been performed on a biomechanical model of the spine and ribcage [14–16]. The geometries of the individual model components were designed separately for females and males, based on diverse anatomical data available in the literature, such as the anatomical dimensions of the vertebral bodies and intervertebral disks per level, as well as the different geometries of the human ribs for female and male [17, 18]. Hence, there are gender-specific biomechanical models for females and males. After the FEM simulations, the patient-specific deformed biomechanical models were then fitted into the 3D body scan image of the patient. To do this in the best possible way, additional information about typical distances and the locations of characteristic reference points is required. This includes, for example, vertebral body to skin distances at different levels and the positions of the vertebral bodies in relation to the transverse body contours. The best fitting biomechanical model in the current 3D body scan image of the patient can then be used to derive the course of the vertebral column (Fig. 1), further described also in the Discussion section. In follow-up examinations, the patient-specific deformed biomechanical model and the spinal course derived from it can be compared with the previous simulation results.

Here, the individual distances from the centre of the vertebral body to the end tip of the spinous process and further along this axis to the skin have been measured from transverse CT slices at thoracic and lumbar vertebral levels. These measurements are in complement to other measures, where the distance from the centre of the vertebral body to the skin has been measured in the sagittal plane [14]. In the case of spinal curvature and vertebral rotation, however, the axis measured here from the centre of the vertebral body through the spinous process end tip to the skin is generally not in the sagittal plane. Further, the locations of the vertebral bodies have been analysed in relation to the patient’s individual transverse body contours and shown together with those of other patients. Although the method presented here was developed based on available CT images from adult patients, these measurements were taken in order to find and evaluate potential trends and characteristics that might be useful for further development of the scoliosis assessment methods that are based on the combination of 3D images from the body scanner and FEM simulations on a self-developed custom biomechanical models [15, 16]. These methods have the potential to become an alternative that does not require the use of ionizing radiation in follow-up examinations and could thus reduce the number of X-rays.

## Methods

### Data

For the present study, twenty axial CT scans of the thorax and abdomen from females and males have been analysed. The ages of the patients ranged from about 54 to 88 years. The CT data were selected from the pool of available images at the radiology department, where patients had a CT scan for other reasons. However, it was confirmed by a senior orthopaedic specialist that the CT incidentally also showed a deformation of the spine. For the purpose of this study, hence, no additional CT scans were performed. Each CT scan was classified by the same senior orthopaedic specialist based on the severity of the spinal curvature into one of three groups: mild, if the Cobb angle was less than 20^∘^; strong, if the Cobb angle was found to be greater than 40^∘^; and medium for all in between [19, 20]. Four out of twenty CT scans thereby fell into the group of mild spinal curvatures, ten into the medium group, and six were classified as strong. Further, with respect to the affected region of the spine, the deformations could be assigned to four regions: (a) thoracic, if the apex of the main curve is located between T2 and T11 vertebrae; (b) thoraco-lumbar, when located around T12 and L1 vertebrae; (c) lumbar, when located in the range from L2 to L5 vertebra; and (d) combined, when it is a combination of thoracic and lumbar lateral curvatures [21, 22]. According to this classification, four of the twenty cases were classified as thoracic spinal curvature, ten as lumbar, three as thoraco-lumbar, and three as combined. Although the methods presented here are based on these available CT images, the software tool developed can be used with any CT images of the vertebral column, regardless of the presence and type of spinal curvature. Here, the measurements were done to find a trend in the position of vertebral bodies relative to the patient’s individual body contour in relation to the severity of spinal deformation.

CT data were made anonymous by the radiology department and all methods and procedures were approved by the ethics committee of the Faculty of Medicine of the Technical University of Munich before starting the study.

### Procedure

**Fig. 2:**
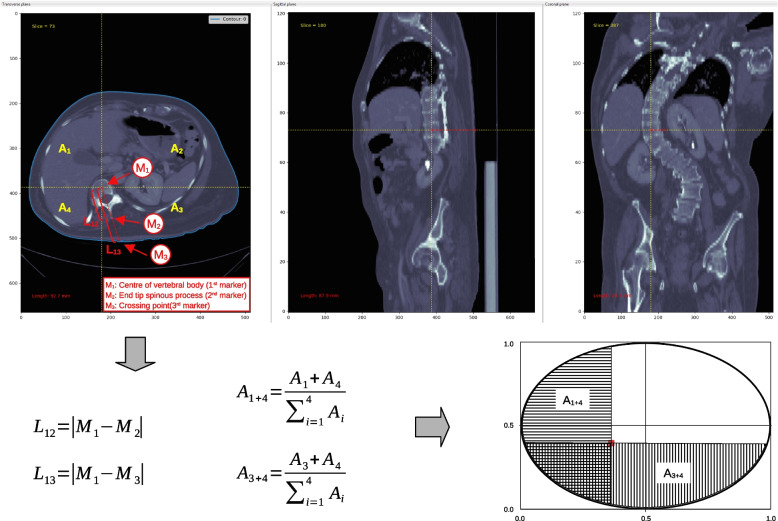
Annotated scheme of the data analysis at the example of a strong spinal deformation seen in computed tomography. At the transverse view, arrows denote the marker positions at the centre of the vertebral body, at the end tip of the spinous process and at the crossing point between the straight through the previous, two markers and the body contour. $A_1$...$A_4$ denote the areas of the four quadrants enclosed by the body contour (blue line), which were used to find the corresponding position inside the ellipse

Self-developed custom software tools were used to measure the individual distances from the vertebral body to the skin and to the end tip of the spinous process on all thoracic and lumbar vertebrae. A total of three digital markers were manually set on each vertebral body (Fig. 2): First, the location of the centre of the vertebral body was marked in the transverse slice closest to the vertical centre of this vertebra. Next, on the same slice, the end tip position of the spinous process, or its projection onto this plane was marked. And last, the position was marked where the straight line through the two previous markers crossed the nearest outer body contour. The euclidean distances ($L_{ij}$) from the first to the second ($L_{12}$) and from the first to the third ($L_{13}$) marker then have been calculated from the corresponding pixel positions and known pixel spacings. These distances are generally not in the sagittal plane in case of spinal deformation, thus complementing previous measurements [14]. Moreover, since the rotations of the vertebral bodies away from the transverse plane were intentionally not considered, the euclidean distance from the first to the second marker ($L_{12}$) does not correspond to the true anatomical length of the respective spinous process.

Further, cross lines through the first marker at each vertebral level – the position closest to the centre of the vertebral body – and parallel to the transverse axes of the laboratory coordinate system were used to divide the area encompassed by the body contour into quadrants. The areas of the four segments, $A_1$...$A_4$, were then normalized with respect to the total area encompassed by the body contour. All areas were calculated by using the Surveyor’s area formula [23] – a mathematical algorithm to determine the area of a simple polygon, whose vertices are given in Cartesian coordinates. Irrespective of the patient’s individual body shape and its body contour the data thus can be compared with other patients. From the normalized areas of the four segments, two aspect ratios were calculated: Back to front and left to right area aspects, respectively. These two aspects were then used to find the corresponding position in a generalized shape, further explained below, for comparison with data from other patients. For presentation, here an ellipse was chosen as a generalized shape, because of its similarity in shape to the natural transverse body contour. However, the ellipse does not need to be fitted to any body contour.

The courses of the transverse body contours have been derived from the CT data by conventional segmentation using the “find_contours” method, based on a marching squares algorithm [24]. This method is part of a collection of algorithms for image processing in Python, called “scikit-image” [25], and returns a list of vertices that form a polygon representing the course of the contour.

**Fig. 3:**
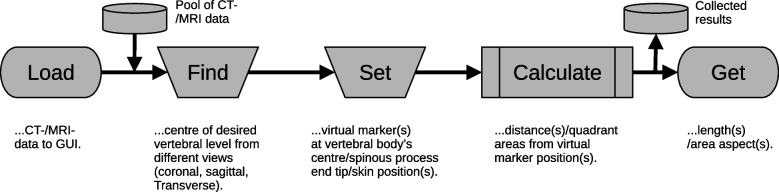
Flowchart of the procedures. Dashed process elements (“Find" and “Set") indicate a currently manual interaction

An annotated scheme at the example of a strong spinal deformation and a flowchart of the procedures described above are shown in Figs. 2 and 3, respectively. At the upper panel of Fig. 2 the screenshot of the internally developed software tool shows three CT image slices perpendicular to the principle axes – a transverse, sagittal, and coronal view (from top left to right). In the transverse view, the three marker positions for the analysis are highlighted by the annotation arrows. The cross hair through the centre of the vertebral body – the first marker position – separates the contour area into the four segments ($A_1$...$A_4$) that have been used to calculate the area aspects $A_{1+4}$ and $A_{3+4}$, respectively. These area aspects were then further used to find the corresponding position of the vertebral body inside the ellipse – the generalized shape chosen here for comparison among different subjects. The position inside the ellipse is defined by the crossing point (red dot) of the two border lines that delimit the horizontally and vertically hatched partial areas with sizes equally to the corresponding area aspects from the body contour.

The crossing lines at all CT views indicate the image cuts seen in the corresponding other two image views for better orientation. In the transverse view, these crossing lines coincide with the contour area separation cross hair. The sagittal and coronal views here mostly help in finding the transverse cuts closest to the vertical centres of the vertebral bodies.

The whole method and software tools were developed using Python programming language (version: 3.8.x).

## Results

**Fig. 4:**
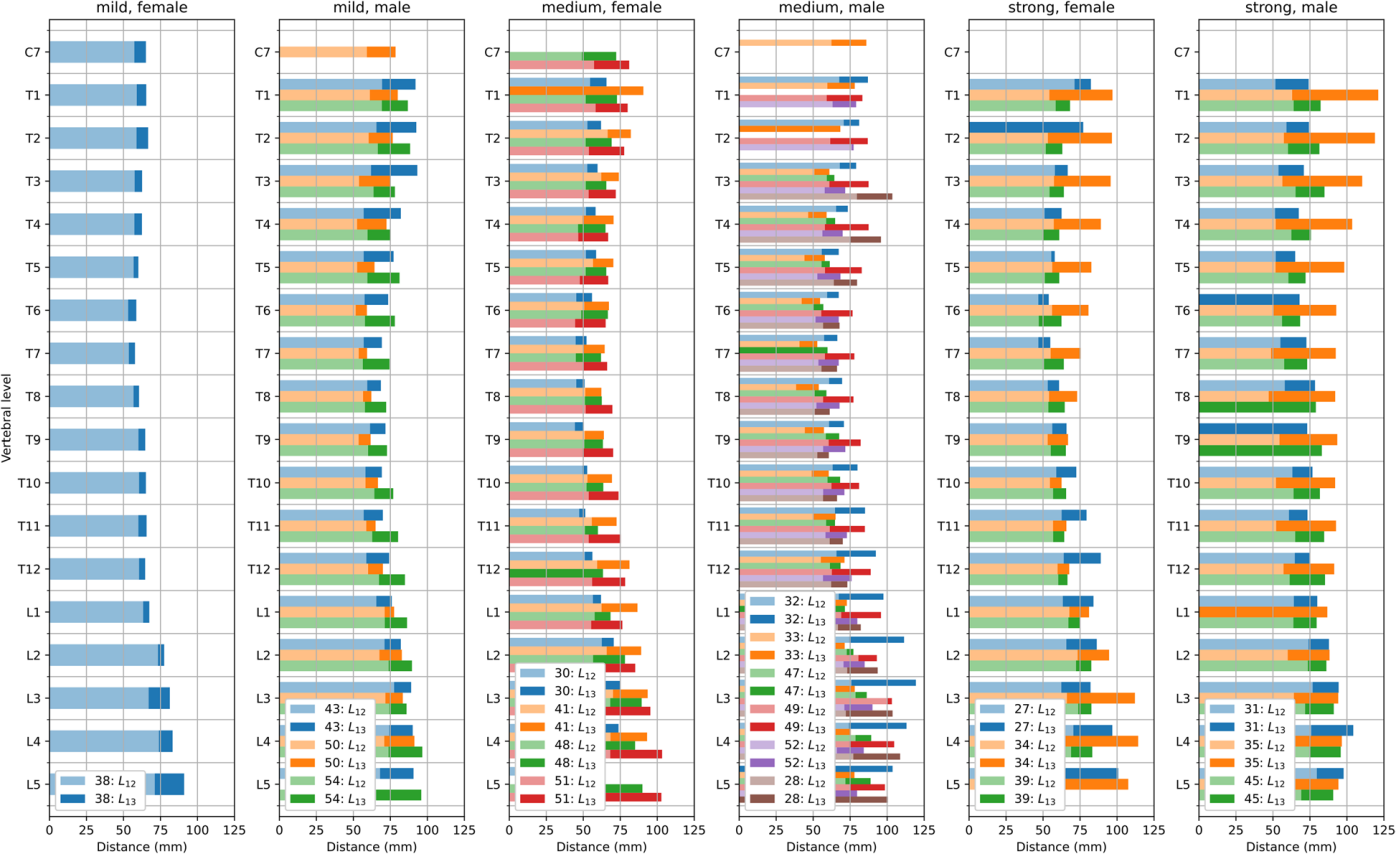
Measured transverse distances between the centres of the vertebral bodies and the spinous processes end tips, $L_{12}$ (light colours), and between the vertebral body centres and the skin, $L_{13}$ (dark colours), at thoracic and lumbar vertebral levels from CT data upon grouping into mild, medium and strong spinal deformation and gender (left to right). Lengths are given in absolute units. Different colours correspond to different patients. (Numerals in the legend denote patient numbers)

**Fig. 5:**
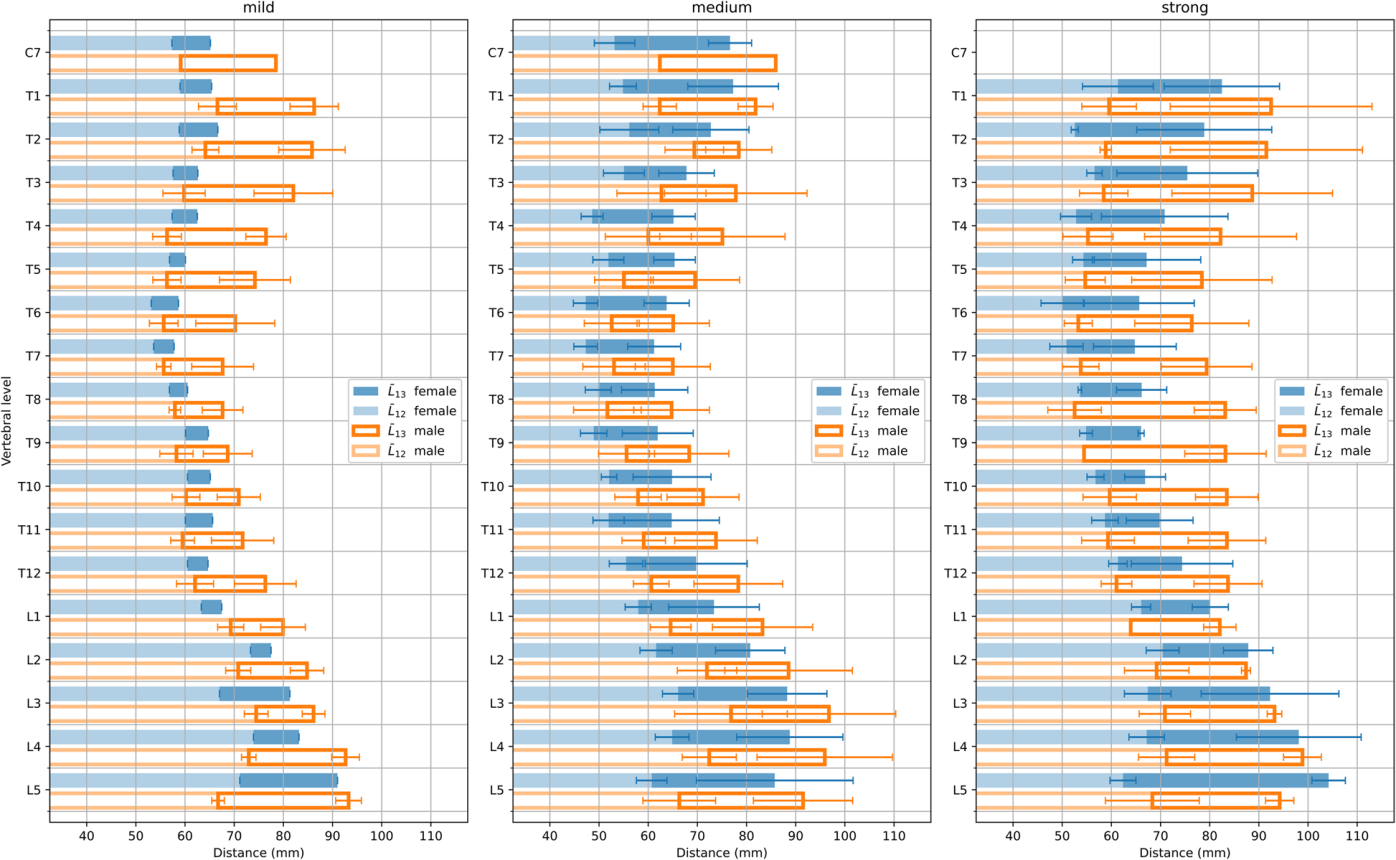
Arithmetic mean and standard deviation of the $L_{12}$ and $L_{13}$ distances per vertebral level, after grouping by gender

Figure 4 shows the transverse euclidean distances from the centre of the vertebral body to the end tip of the spinous process, $L_{12}$ (light colour), and from the vertebral body to the skin, $L_{13}$ (dark colour), respectively, for all thoracic and lumbar vertebral levels. The distances are given in absolute units. Further, data were grouped according to the severity of the patient’s spinal curvature from mild to strong, and by gender (left to right). If no clear end tip position of the spinous process could be identified, only the distance from the centre of the vertebral body to the skin is given (dark colour). Figure 5 shows the corresponding arithmetic mean values and their standard deviations of the lengths $L_{12}$ and $L_{13}$. Table 1 lists the corresponding arithmetic mean values and their standard deviations per level (top) and region (bottom), with no separation by gender.

**Table 1:** Arithmetic mean and standard deviation (SD) per vertebral level (top) and region (bottom) of the $L_{12}$ and $L_{13}$ lengths in absolute units of mm, shown in Fig. 4

**Level:**	**Mild:**				**Medium:**				**Strong:**			
	$L_{12}$ [mm]		$L_{13}$ [mm]		$L_{12}$ [mm]		$L_{13}$ [mm]		$L_{12}$ [mm]		$L_{13}$ [mm]	
	Mean	(SD)	Mean	(SD)	Mean	(SD)	Mean	(SD)	Mean	(SD)	Mean	(SD)
C7	58	(0.9)	72	(6.7)	56	(5.5)	80	(5.7)				
T1	65	(4.7)	81	(10.0)	59	(4.8)	80	(7.4)	60	(6.5)	87	(17.4)
T2	63	(3.3)	81	(10.1)	62	(8.9)	76	(7.8)	56	(3.2)	85	(18.0)
T3	59	(3.9)	77	(10.9)	60	(8.4)	74	(12.8)	58	(3.8)	82	(16.7)
T4	57	(2.6)	73	(7.0)	55	(8.9)	71	(11.3)	54	(4.4)	77	(15.3)
T5	56	(2.5)	71	(8.7)	54	(5.3)	68	(7.8)	54	(3.3)	73	(13.9)
T6	55	(2.7)	67	(8.6)	50	(5.3)	65	(6.4)	51	(4.1)	71	(12.6)
T7	55	(1.6)	65	(6.9)	50	(5.8)	63	(7.1)	52	(3.8)	72	(11.5)
T8	58	(1.1)	66	(4.7)	51	(5.7)	63	(7.5)	53	(3.5)	75	(10.3)
T9	59	(3.0)	68	(4.6)	53	(5.7)	66	(8.3)	55	(1.2)	75	(10.4)
T10	60	(2.5)	69	(4.6)	56	(4.8)	69	(8.2)	58	(4.3)	75	(9.9)
T11	60	(2.1)	70	(6.1)	56	(5.3)	70	(10.0)	59	(4.3)	77	(10.1)
T12	62	(3.3)	73	(7.4)	59	(4.3)	75	(10.5)	61	(2.6)	79	(10.0)
L1	68	(3.5)	77	(6.7)	62	(4.8)	79	(10.9)	65	(1.8)	81	(3.6)
L2	71	(2.5)	83	(4.3)	68	(7.2)	85	(11.6)	70	(5.2)	88	(3.6)
L3	73	(3.9)	85	(2.9)	73	(10.5)	93	(12.4)	69	(5.3)	93	(10.0)
L4	73	(1.3)	90	(4.8)	69	(6.0)	93	(13.1)	69	(5.2)	98	(9.4)
L5	68	(2.3)	93	(2.4)	64	(6.6)	90	(12.6)	66	(8.1)	98	(5.8)
**Mean:**	$\overline{L_{12}}$ [mm]		$\overline{L_{13}}$ [mm]		$\overline{L_{12}}$ [mm]		$\overline{L_{13}}$ [mm]		$\overline{L_{12}}$ [mm]		$\overline{L_{13}}$ [mm]	
all	62	(2.6)	76	(6.5)	59	(6.3)	76	(9.5)	60	(4.2)	81	(11.1)
T2...T11	58	(2.5)	71	(7.2)	55	(6.4)	68	(8.7)	55	(3.6)	76	(12.9)
T12/L1	65	(3.4)	75	(7.0)	60	(4.6)	77	(10.7)	63	(2.2)	80	(6.8)
L2...L5	71	(2.5)	88	(3.6)	68	(7.6)	90	(12.5)	69	(6.0)	94	(7.2)

Accordingly, Table 2 shows the arithmetic mean values and their standard deviations per region, after grouping by the range of spinal deformation and with no separation by gender.

**Table 2:** Arithmetic mean and standard deviation (SD) of the $L_{12}$ and $L_{13}$ lengths in absolute units of mm, after grouping by range of scoliosis

Level:	Thoracic:				Thoraco-lumbar:				Lumbar:				Combined:			
	$\overline{\boldsymbol{L}_{\boldsymbol{12}}}$ [mm]		$\overline{\boldsymbol{L}_{\boldsymbol{13}}}$ [mm]		$\overline{\boldsymbol{L}_{\boldsymbol{12}}}$ [mm]		$\overline{\boldsymbol{L}_{\boldsymbol{13}}}$ [mm]		$\overline{\boldsymbol{L}_{\boldsymbol{12}}}$ [mm]		$\overline{\boldsymbol{L}_{\boldsymbol{13}}}$ [mm]		$\overline{\boldsymbol{L}_{\boldsymbol{12}}}$ [mm]		$\overline{\boldsymbol{L}_{\boldsymbol{13}}}$ [mm]	
	Mean	(SD)	Mean	(SD)	Mean	(SD)	Mean	(SD)	Mean	(SD)	Mean	(SD)	Mean	(SD)	Mean	(SD)
all	60	(3.3)	79	(11.6)	61	(3.6)	76	(5.8)	59	(6.1)	75	(9.0)	61	(4.2)	82	(8.5)
T2...T11	57	(3.3)	76	(14.9)	57	(3.8)	71	(5.9)	54	(5.9)	69	(9.0)	58	(4.3)	73	(8.3)
T12/L1	62	(1.0)	77	(9.3)	64	(4.0)	78	(6.0)	61	(4.6)	75	(8.8)	65	(2.2)	85	(9.0)
L2...L5	70	(5.8)	88	(4.3)	69	(2.6)	87	(5.0)	69	(7.7)	89	(10.2)	68	(4.3)	104	(9.3)

Irrespective of the severity of the spinal curvature, the transverse euclidean distances from the centres of the vertebral bodies to the end tips of the spinous processes, $L_{12}$ (cf. light coloured horizontal bars), follow a physiological trend. That is, they are maximal in the lumbar region (L2...L5) and decrease towards their minima around T7 level before they slightly increase again towards the upper thoracic/lower cervical region (T1...T4). Accordingly, the distances from the centres of the vertebral bodies to the skin, $L_{13}$ (cf. dark coloured horizontal bars) follow a similar trend, although their increase towards the upper thoracic/lower cervical region appears slightly more pronounced: $\frac{\overline{L_{12}}\text {(T1...T4)}}{\overline{L_{12}}\text {(T5...T9)}} < \frac{\overline{L_{13}}\text {(T1...T4)}}{\overline{L_{13}}\text {(T5...T9)}}$ for mild, medium and strong, where $\overline{L_{ij}}$ denotes the regional arithmetic mean. When grouped by gender, the arithmetic mean values are often smaller for females than for males (cf. Fig. 5).

Further, the variations among different patients of the vertebral body to skin distances are larger and much increase with the severity of the spinal curvature in comparison to the corresponding vertebral body to spinous processes distances. That is, $\text {SD}(\overline{L_{13}}) > \text {SD}(\overline{L_{12}})$ for mild medium and strong, and generally $\text {SD}^{\text {Mild}}(\overline{L_{13}})< \text {SD}^{\text {Medium}}(\overline{L_{13}}) < \text {SD}^{\text {Strong}}(\overline{L_{13}})$, but for the lumbar region in case of strong, with $\text {SD}(\overline{L_{1i}})$ being the corresponding standard deviation (SD) of $\overline{L_{1i}}$ (cf. Table 1 bottom). Similarly, the standard deviation of the arithmetic mean of the vertebral body to skin distances appears to be maximal in the region that corresponds to the range of spinal curvature. That is, in case of thoracic curvature $\text {SD}^{\text {T2...T11}}(\overline{L_{13}})> \text {SD}^{\text {T12/L1}}(\overline{L_{13}}) > \text {SD}^{\text {L2...L5}}(\overline{L_{13}})$ and accordingly $\text {SD}^{\text {T12/L1}}(\overline{L_{13}}) > \text {SD}^{\text {T2...T11}}(\overline{L_{13}})$ & $\text {SD}^{\text {T12/L1}}(\overline{L_{13}}) > \text {SD}^{\text {L2...L5}}(\overline{L_{13}})$ in case of thoraco-lumbar, and $\text {SD}^{\text {L2...L5}}(\overline{L_{13}}) > \text {SD}^{\text {T12/L1}}(\overline{L_{13}})$ & $\text {SD}^{\text {L2...L5}}(\overline{L_{13}}) > \text {SD}^{\text {T2...T11}}(\overline{L_{13}})$ (cf. Table 2).

**Fig. 6:**
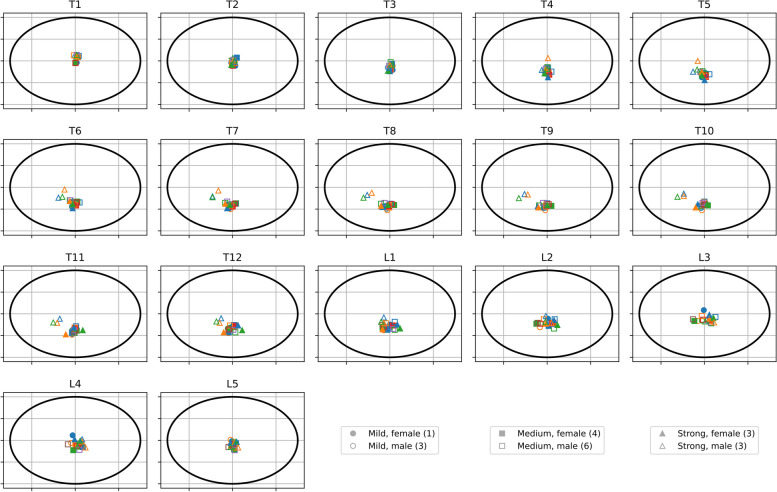
Positions of the thoracic and lumbar vertebral bodies normalized to the patient’s individual body contours derived from CT data. Different symbols represent different severities of spinal deformation – mild (circle), medium (square) and strong (triangle). Filled and open symbols refer to female and male patients. Different colours per symbol correspond to different patients with the same severity of spinal deformation. (Numerals in the legend refer to the number of patients in this group)

Figure 6 shows the distribution of the centres of the vertebral bodies from all patients at all thoracic and lumbar vertebral levels. The three different symbols – circle, triangle, and square – are associated with the three classes of severity of the spinal deformation. Filled and open symbols refer to female and male patients, while different colours per symbol correspond to different patients within the same class of severity.

The location of the cluster of the centres of the vertebral bodies varies with vertebral level. While at the upper thoracic and lower lumbar levels the vertebral body positions accumulate near the centre of the ellipse, the cluster shifts towards the middle of the lower/back half section at levels T7...T10. Strong spinal curvatures further show a clear lateral spread. With increasing spread their positions also seem to shift towards the centre line between back and front. The data do not show any clear trend or difference between females and males.

## Discussion

In the present study, software tools and methods are presented to measure the distances between the vertebral body and the end tip of the spinous process, and between the vertebral body and the skin from transverse CT images. Furthermore, normalization with respect to the area enclosed by the individual transverse body contour allowed to display the location of the vertebral bodies in relation to a generalized transverse body contour shape along with those of other patients, regardless of their body shape and size.

To our knowledge, this is the first study analysing the vertebral body to skin distances in the transverse plane along the axis through the spinous process end tip and showing the centre position of the vertebral bodies normalized to the area encompassed by the transverse body contour from transverse CT images. The present measurements along the axis from the vertebral body through the spinous process end tip are thus different from other measurements [14], where the distances have been measured along the intersection of the transverse and sagittal plane. In case of spinal deformation, however, the axis from the vertebral body through the spinous process end tip is not collinear to the sagittal plane. Several anatomical data are available in the literature for the vertebral body structures [17, 18]. The present study aims to investigate the localization of vertebral bodies within body contours in scoliosis using CT images. The final aim is to develop a non-invasive scoliosis assessment method in which a biomechanical thoracic spine model [15] based on anatomical data will be optimally integrated into body contours. The present findings might help to optimize the positioning of the model into the transverse cross sections of the torso.

The relatively small variations in the distances between the vertebral bodies and the end tips of the spinous processes in different patients, represented by the standard deviation (SD) of the corresponding $L_{12}$ mean (cf. Fig. 5, Table 1), indicate that this measured distance seems to be similar in adult patients with different ages and within the same gender group. However, no clear evidence that this distance is independent of age could be given, as the patients were randomly selected and the number of patients available for this study does not allow for further statistics after grouping by age. Furthermore, as the images were anonymized, no further information about the individual body shapes is available than can be seen in the CT images themselves. Since these distances were measured in transverse view (cf. Fig. 2), they do not necessarily correspond at all vertebral levels to the distances between the centre of the vertebral bodies and the spinous processes end tips of the same vertebral bodies. The variation of the distances between the vertebral bodies and the skin is much larger than the variation of the distances between the vertebral bodies and spinous processes end tips, i.e. generally $\text {SD}(L_{13}) > \text {SD}(L_{12})$, since the vertebral body to skin measures include also the individual thicknesses of the skin tissue, fat and musculature. Moreover, the variation of this distance appears to increase with the severity of the spinal deformation (cf. Table 1), since in some cases, rotation of the vertebral bodies about the vertical axis may additionally be involved. Accordingly, the arithmetic mean of the variations is maximal at its corresponding range of scoliosis (cf. Table 2). Methodically, the measured distance between the vertebral body and the skin is therefore also affected by the rotation of the vertebral body around the vertical body axis. Since the length of the straight from the vertebral body through the end tip of the spinous process was measured, this distance increases with increasing rotation of the vertebral body. These distances seem not to be correlated with the range of scoliosis, though, but on average increase from top – thoracic – to bottom – lumbar – region in either case of grouping (cf. $\overline{L_{13}}$ in Tables 1 & 2). However, a direct comparison with other results is complicated by the fact that, to our knowledge, these particular distances have not yet been measured anywhere else.

Further, CT images are taken in the supine position, while the patient is standing upright for the body scan. This is one of the limitations of the present study because the spine bears more load in the standing position while the spine is unloaded in the supine position. However, previous studies showed that Cobb angles measured in supine positions were linearly correlated with the Cobb angles measured in standing positions  [26]. Therefore, the present measured distances could have been underestimated but might be correlated to scan data from standing upright position with an acceptable degree of accuracy for the application presented here [27–29]. However, the correlation has not been investigated here, since CT images and body scans were not available from the same person. Furthermore, the shape of individual transverse body contours may vary in the supine and standing positions due to the influence of muscles and fat tissues.

The method of presenting the positions of the vertebral bodies as a function of the normalized transverse body area quadrants enables the plotting of the locations with those from other patients, regardless of their individual body shape and contour, and to identify potential clusters. The distinct lateral spread of some of the locations of the vertebral bodies at certain vertebral levels is associated with a pronounced spinal deformation. In these cases, the accompanying shift of the location of the vertebral bodies towards the mid-line between back and front is due to the distinct rotation of the vertebral bodies around the vertical body axis and a clear asymmetry in the transverse body contour, due to the natural rib hump with posterior elevation at the convex side.

For female patients, the position of the thoracic vertebra is systematically biased by the proportion of the transverse area of the female breast. Hence, the positions of the thoracic vertebrae are slightly shifted towards the back at the levels of the female breast. This systematic effect, however, is expected to be rather small, as the fraction of the female breast of the transverse area is minor. An analysis of the location of the vertebral bodies did not show any clear trend upon gender grouping (cf. Fig. 6) and thus is in agreement with the prior assumption. For the same reason, a large abdomen also slightly affects the normalized positions of the vertebrae, irrespective of gender.

Intra- and inter-observer variability would be expected to be rather small, but mostly could have affected the positioning of the markers and the selection of the transverse slices closest to the vertical centre of the vertebral bodies. However, symmetry detection is an efficient, salient human visual property [30] and thus the precision of the human positioning of the markers can be expected to be high. For a similar reason, the observer variability in the selection of transverse slices is also expected to be rather small at the present step size of 5 mm along the CT scan direction. With potentially smaller step sizes, the differences among slices are getting smaller, which in turn reduces the potential error in visual selection of the centre slice.

Measurements of distances and angles of anatomical structures are common in orthopaedic and radiological practice but can be difficult on three-dimensional (3D) stacked images, such as CT and MRI, because different parts of the structure are seen on different images, contrary to plain X-ray. Various tools thus have been developed, for instance by Maizlin and Vos, to measure the Cobb’s angles and others on MRI and CT [31]. Recently also machine learning gained importance in 3D image analysis [32] and in getting anatomical information from CT and MRI images [33]. Further, machine learning is used in this field also for localization, segmentation [34], and others.

The results of the present study, however, could be of interest to health professionals and medical scientists. Detailed measurements of the anatomy are important in various fields. These measurements may help to further understand the pathomechanisms of spinal deformations [35] or to identify reference points in X-ray examination that could be used for reconstruction of a 3D spinal model [36, 37]. Or in another example, they could be used in combination with other morphological data and anatomical information to improve the prediction of 3D spinal alignment from external shape [38], or the fitting of individually distorted biomechanical models of the vertebral column to body scanner images in the course of developing scoliosis assessment methods that do not require the use of ionizing radiation during follow ups [16]. Although young patients who show signs of scoliosis and are particularly affected by regular X-rays usually have idiopathic or neurogenic scoliosis, the methods presented here can also be transferred to other types of scoliosis, such as muscular, degenerative, and congenital scoliosis at all ages.

A schematic representation of the potential use of the current results in combination with data from other studies [15–17] was indicated in the Background section and depicted in Fig. 1. There, one of the challenges appears to be an automated fitting of an individually distorted biomechanical model (Fig. 1b.) inside the patient’s 3D body scan (Fig. 1a./d.). Data from the present study, like the positions of the vertebral bodies and typical distances from the skin could help to confine the number of degrees of freedom in the fitting algorithm at certain vertebral levels (Fig. 1c.). Furthermore, also health professionals and in particular orthopaedic specialists could profit from this internally developed, user friendly software that displays the anatomical structures in high detail from different perspectives – coronal, sagittal, and transversal – and provides different measurement tools that could be easily further developed according to the specialists desires. Despite major achievements in identifying, segmenting, and localizing anatomical structures by artificial intelligence and machine learning, visual inspection of the individual CT images and diverse measuring tools are important for medical professionals to provide proper individual treatment.

In the future, the present principle study will be extended to more cases and the marker positions will additionally be used to analyse the rotation of the vertebral bodies. Also, the positioning of the markers could potentially be automated, using (semi-) automatic localization tools to find characteristic points in 3D volumes. Further, the measured distances could be corrected for different body sizes, using an approach similar to that used to correct the location of the centre of the vertebral within an ellipse.

## Conclusion

Distances between the centres of the vertebral bodies and the spinous processes end tips, and from the vertebral bodies to the skin have been measured from transverse CT images at all thoracic and lumbar levels from patients with various severe spinal deformation. The distances between the vertebral bodies and the spinous process end tips appeared to be similar at corresponding vertebral levels after grouping by gender. However, trends could be found as a function of the vertebral level. Further, the vertebral body centers positions could be displayed at thoracic and lumbar vertebral levels across different patients by transverse body contour area normalization. Again, the first trends as a function of vertebral level and upon grouping into mild, medium, and strong spinal deformation were found within the limited number of analysed CT scanes, which could further be used in context with biomechanical modeling of patient’s individual spinal deformation in scoliosis assessment.

## Data Availability

The data analyzed for the current study are available from the corresponding author on reasonable request.

## Abbreviations

3D: Three-dimensional
CT: Computed tomography
FEM: Finite-element methods
MRI: Magnetic resonance imaging
SD: Standard deviation
WHO: World health organization
